# Supplementary material for: Development of a computer-aided design software for the quantitative evaluation of aesthetic damage
Source: PLoS One. 2019 Dec 18;14(12):e0226322. doi: 10.1371/journal.pone.0226322 (PMC6919621; doi:10.1371/journal.pone.0226322)
Supplement: S1 Code site — 1. (ZIP) [file pone.0226322.s001.zip › [EESC_jr][Projeto]Relatorio_07_21/reports/templates_zip/gentelella-master/vendors/bootstrap-wysiwyg/examples/clear-formatting.html]

A tiny, opensource, Bootstrap WYSIWYG rich text editor


# Simple Editor with Toolbar

- Huge
- Normal
- Small

Add

Submit
